# 1379. Vaccination Rates among Liver Transplant Recipients at a Tertiary Care Hospital in Newark, NJ

**DOI:** 10.1093/ofid/ofab466.1571

**Published:** 2021-12-04

**Authors:** Tilly Varughese, Michael Song, Joachim Sackey

**Affiliations:** 1 Rutgers New Jersey Medical School, Newark, New Jersey; 2 Rutgers School of Public Health, Newark, NJ

## Abstract

**Background:**

Transplant candidates and recipients are at increased risk of infectious complications of vaccine-preventable diseases due to their longstanding immunosuppressive regimens. We assessed the rates of vaccination in our liver transplant patients at University Hospital (UH) in Newark, NJ.

**Methods:**

Retrospective chart-review including patients > 18 years old who underwent liver transplantation at UH for a 3-year period from 01/01/2017 to 07/20/2020. Data collected included demographics, clinical outcomes, eligibility and receipt of vaccinations before and after transplantation, protection titers after administration of hepatitis vaccinations and presence of an ID outpatient consultation. We looked at the following receipt of vaccinations: Prevnar-13, Pneumovax-23, Influenza, TDaP, Shingrix, Varivax, Havrix and Engerix/Heplisav. Characteristics of study participants was analyzed using descriptive statistics and Chi-Square/Fisher’s Exact tests were used to test associations.

**Results:**

119 unique medical charts were reviewed and no patients were excluded. Of those patients who were eligible to receive Hepatitis A vaccination, only 44.8% were documented to receive vaccination and of those eligible to receive Hepatitis B vaccination, only 47.8% received it. Influenza vaccination pre-transplantation was 46% and 66.1% in post-transplant recipients. For the other vaccinations, during the pre-transplant period, 17.6 % of patients received Prevnar-13, 36.1% Pneumovax-23 and 20.2% TDaP and 26.1% received Shingrix. Patients who had ID consultation were significantly more likely to receive appropriate Hepatitis A and Hepatitis B vaccinations (p values 0.026 and 0.005).

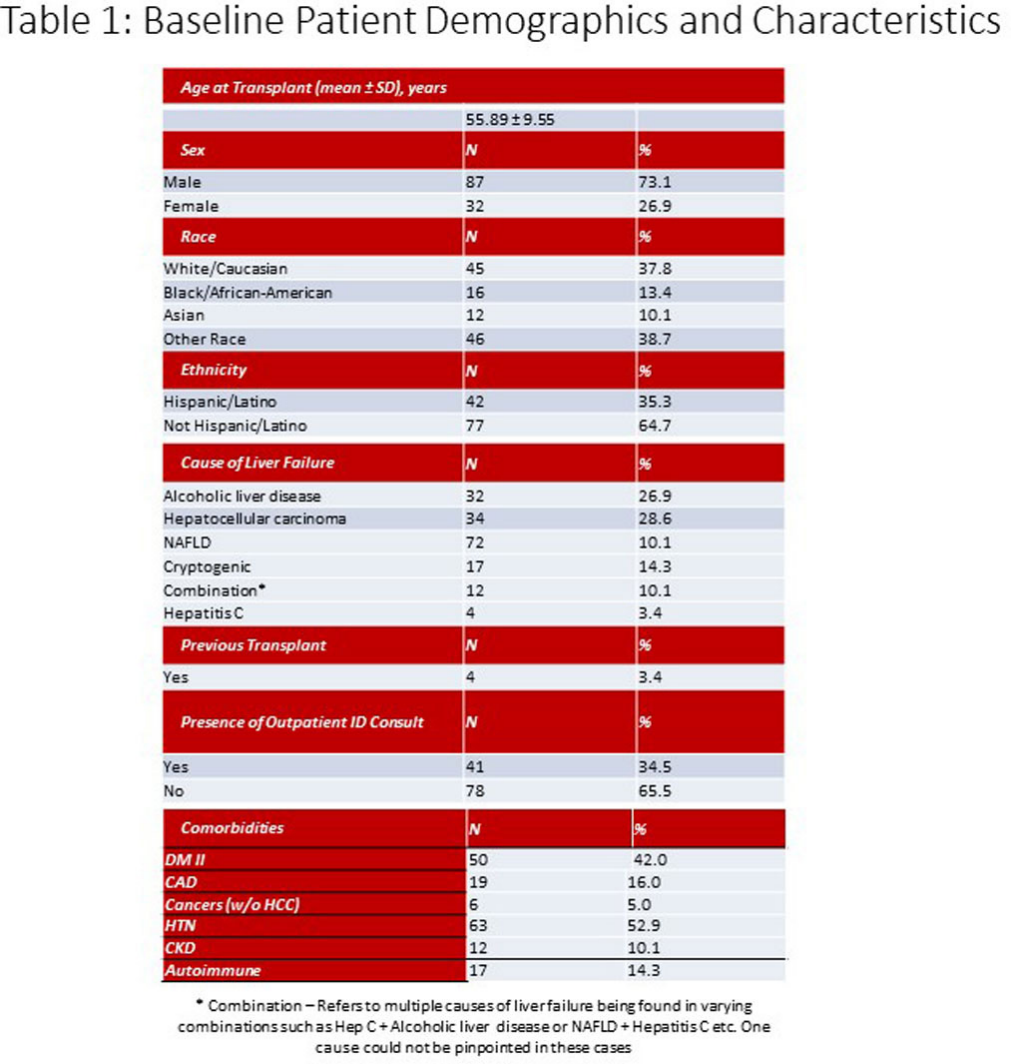

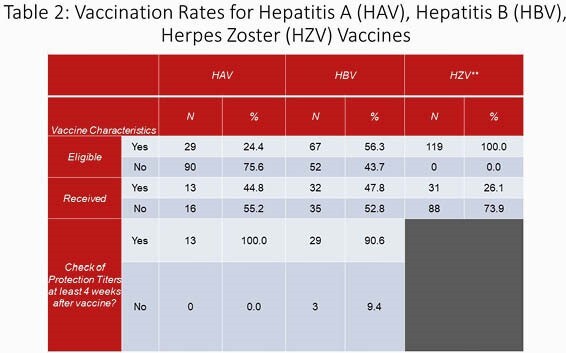

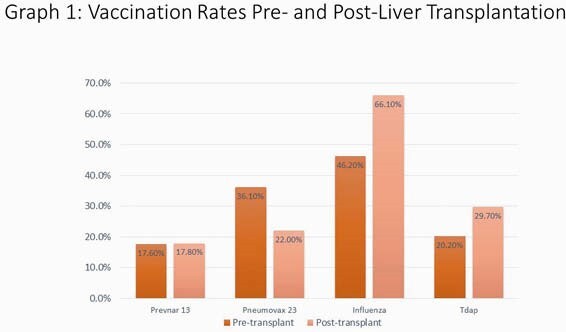

**Conclusion:**

We are not meeting national vaccination standards set by the American Society of Transplantation (AST) for optimal vaccination in this population. Our study can inform of possible solutions to increase vaccination rates in this population such as the simple addition of a smartphrase within EMR notes to remind providers to order appropriate vaccinations and eventually, a more long term solution of creation of a dedicated vaccination clinic and/or routine ID pre-transplant evaluations for all transplant candidates.

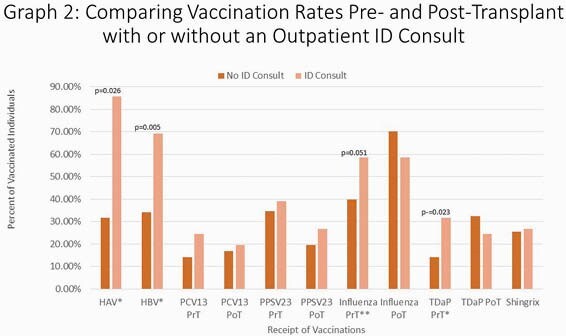

**Disclosures:**

**All Authors**: No reported disclosures

